# Silencer of Death Domains Controls Cell Death through Tumour Necrosis Factor-Receptor 1 and Caspase-10 in Acute Lymphoblastic Leukemia

**DOI:** 10.1371/journal.pone.0103383

**Published:** 2014-07-25

**Authors:** Adam Cisterne, Rana Baraz, Naveed I. Khan, Robert Welschinger, Jordan Basnett, Carina Fung, Helen Rizos, Kenneth F. Bradstock, Linda J. Bendall

**Affiliations:** 1 Centre for Cancer Research, Westmead Millennium Institute, University of Sydney, Sydney, New South Wales, Australia; 2 Blood and Marrow Transplant Service, Department of Haematology, Westmead Hospital, Sydney, New South Wales, Australia; German Red Cross Blood Service Frankfurt, Germany

## Abstract

Resistance to apoptosis remains a significant problem in drug resistance and treatment failure in malignant disease. NO-aspirin is a novel drug that has efficacy against a number of solid tumours, and can inhibit Wnt signaling, and although we have shown Wnt signaling to be important for acute lymphoblastic leukemia (ALL) cell proliferation and survival inhibition of Wnt signaling does not appear to be involved in the induction of ALL cell death. Treatment of B lineage ALL cell lines and patient ALL cells with NO-aspirin induced rapid apoptotic cell death mediated via the extrinsic death pathway. Apoptosis was dependent on caspase-10 in association with the formation of the death-inducing signaling complex (DISC) incorporating pro-caspase-10 and tumor necrosis factor receptor 1 (TNF-R1). There was no measurable increase in TNF-R1 or TNF-α in response to NO-aspirin, suggesting that the process was ligand-independent. Consistent with this, expression of silencer of death domain (SODD) was reduced following NO-aspirin exposure and lentiviral mediated shRNA knockdown of SODD suppressed expansion of transduced cells confirming the importance of SODD for ALL cell survival. Considering that SODD and caspase-10 are frequently over-expressed in ALL, interfering with these proteins may provide a new strategy for the treatment of this and potentially other cancers.

## Introduction

Acute lymphoblastic leukemia (ALL) is the most common malignancy in children and although remission is almost always attained, up to 20% of children will relapse, with subsequent poor prognosis [1]. Adult patients have a worse outlook, with more than half relapsing [2]. Current management of ALL in both children and adults is dependent on treatment with multiple chemotherapy drugs, such as corticosteroids and vincristine, which induce apoptosis in the leukemia cells. However, resistance to drug-induced apoptosis is a common problem, and there is an urgent requirement for new drugs with efficacy against leukemic cells in ALL.

Apoptosis can be mediated via the extrinsic death receptor-mediated pathway, or the intrinsic mitochondrial pathway. Cell death is ultimately mediated by activation of effector caspases including caspase-3 and -7. However, the initiator caspases differ between the two pathways, with caspases-8 and -10 being involved in the extrinsic, and caspase-9 in the intrinsic pathway [3]. Commitment to the intrinsic pathway occurs when cytochrome c is released from mitochondria as a result of mitochondrial outer membrane permeabilization (MOMP) [4]. This results in the formation of the apoptosome by recruitment of APAF-1 and pro-caspase-9 [5]. Here caspase-9 is activated by cleavage and in turn activates the effector caspases. The extrinsic death pathway is initiated by the binding of death ligands, such as tumour necrosis factor alpha (TNF-α), TNF-related apoptosis inducing ligand (TRAIL) or FasL to their respective cell surface death receptors, tumour necrosis factor receptor 1 (TNF-R1), death receptors (DR) 4 or 5, and Fas. Oligomerization of the death domains in the cytoplasmic regions of these receptors is the initial event in signalling through these receptors. This can be inhibited by silencer of death domain (SODD), alternatively known as BCL2-associated athanogene 4 (BAG4) in the case of TNF-R1, Fas and DR3 [6], [7]. Once oligomerization has occurred, binding of the adaptor molecules, TNF-R1-associated death domain protein (TRADD) or Fas-associated protein with death domain (FADD), depending on the receptor involved, and pro-caspases-8 or -10 produces the death-inducing signaling complex (DISC) [8]. In some cells activation of caspases-8 or -10 within the DISC is sufficient to facilitate direct activation of effector caspases and cell death, while in others linkage to the intrinsic pathway is needed. This occurs by caspase-8 or -10-mediated cleavage of Bid and induction of MOMP [9].

Despite expression of surface death receptors, including TNF-R1, Fas and TRAIL-R1 and R2, cells from a significant proportion of ALL patients are resistant to ligand-induced apoptosis when exposed to TNF-α, FasL or TRAIL [10]–[12]. The reasons for this are unclear but are thought to involve alterations to death receptor signalling pathways. The specific role of caspase-10 in the induction of cell death is not clear and in most settings it takes a subordinate role to caspase-8. Mice naturally lack caspase-10 [13] and in humans it can substitute for caspase-8 in certain cell types [14]. However, mutations in *CASP10* are associated with type II autoimmune lymphoproliferative syndrome suggesting it has a significant role in lymphoid cells [15]. Caspase-10 is highly expressed in lymphoid cells and can be mutated in lymphoid malignancies [16], including in ALL, although this appears to be rare [17]. Activity of caspase-10 has been implicated in the response to a number of chemotherapeutic agents including etoposide, doxorubicin, arsenic trioxide and paclitaxel [18]–[20]. This can be mediated by p53-dependent, or histone-H3 acetylation-dependent modulation of the *CASP10* locus [18].

We have previously reported that the nitric oxide donating non-steroidal anti-inflammatory drug (NO-NSAID) *para*-NO-aspirin (*para*-NO-ASA) has anti-leukemic activity against human ALL by induction of apoptosis in association with suppression of NF-κB signalling [21]. NO-NSAID drugs consist of traditional NSAID to which an NO-donating moiety is covalently attached via an aromatic or aliphatic spacer [22]. These compounds demonstrate substantial anti-cancer activity and are up to 20 fold more potent than the parental NSAID [23]. *para*-NO-ASA is the best characterized NO-NSAID and *para*-NO-ASA demonstrated a several hundred-fold increase in potency compared with aspirin [24].

In this study we examined the mechanisms involved in *para*-NO-ASA induced apoptosis. We found that *para*-NO-ASA resulted in caspase-10 dependent cell death. Although associated with caspase-10 binding to TNF-R1, there was no evidence to suggest that cell death was ligand dependent. Cell death was associated with suppression of SODD, suggesting that SODD provides protection from death receptor induced death. Agents that specifically suppress SODD may be of therapeutic benefit for the treatment of this disease. Furthermore SODD is overexpressed in a number of malignancies including ovarian and pancreatic cancers [25], [26] and suppression of SODD has recently been associated with the induction of cell death in melanoma [27], suggesting that modulation of SODD may have broader application in cancer treatment.

## Experimental Procedures

### Chemicals and reagents

*para*-NO-ASA [2-(acetyloxy)benzoic acid 4-(nitrooxymethyl)-phenyl ester] was purchased from NicOx (Sophia Antipolis, France). The inhibitors of caspase-8 (Z-IETD-FMK) and caspase-9 (Z-LEHD-FMK), Fix/Perm and Perm/Wash solutions, mouse anti-human TNF-α PE, annexin V FITC and AlexaFluor 647, and 7AAD were obtained from Becton Dickinson (North Ryde, NSW, Australia). Tetramethylrhodamine methyl ester (TMRM) was purchased from Molecular Probes (Invitrogen, Grand Island, NY, USA), N-acetyl cysteine (NAC) from Sigma-Aldrich (St Louis, MO, USA), the InnoCyte Flow Cytometric Cytochrome C release Kit from Calbiochem (San Diego, CA, USA) and the Caspase-8 (BF4100) and caspase-9 (BF10100) activity assays were purchased from Bioscientific (Sydney, Australia). The following antibodies to human proteins were purchased: mouse anti-β-actin (Sigma-Aldrich); rabbit anti-SODD (Chemicon, Boronia, VIC, Australia); rabbit anti-PUMA, rabbit anti-Bcl-X_L_ Alexa Fluor-488, rabbit anti-BIM, rabbit anti-XIAP, rabbit anti-TNF-R1, rabbit anti-Bid, rabbit anti-cleaved caspase-9, rabbit anti-cleaved caspase-8, rabbit anti-full length caspase-10 (Cell Signaling Technologies, Boston, MA, USA); mouse anti-Noxa (Abcam, Cambridge, MA, USA). Goat anti-mouse Alexa Fluor-488 was purchased from Invitrogen. Human recombinant TNF-α was purchased from Millipore (Nth Ryde, NSW, Australia). Caspase-10 inhibitor AEVD-FMK was purchased from Biovision (Mountain View, CA, USA) and FAM Caspase-10 kit from AbD Serotec (Raleigh, NC, USA). Primers for PCR amplification were synthesized by Proligo (Lismore, NSW, Australia) or Sigma Aldrich.

### Cells

Pre-B ALL cell lines NALM6 (DSMZ Braunschweig, Germany), Reh (ATCC, Manassas, VA, USA) and LK63 (gift of Prof A. Boyd, QMIR, Australia) [28], the Burkitt's lymphoma lines Raji and Daudi and T-ALL lines Jurkat and CEMT4 (ATCC) were cultured in RPMI medium with 10% fetal calf serum. The stromal-dependent cells were grown in RPMI with 10% fetal calf serum, on confluent layers of either a human bone marrow stromal cell line transformed with hTERT, here termed hTERT.BMS, (kind gift of Dr D. Campana, Memphis TN, USA) [29] or bone marrow stromal cells prepared as previously described [30].

Leukemic blasts were isolated from the bone marrow samples from five patients with B cell progenitor ALL and cryopreserved as previously described [31]. Samples were collected following informed written consent from the donor or guardian as appropriate and with ethics approval from the Sydney West Area Heath Service Human Research Ethics Committee. Details for 0407 cells have been previously published [32]. The details of the stromal-dependent lines, including the patient characteristics have been previously published [33], [34]. A summary of the clinical information for all patient-derived samples and cell lines used in this study has been provided in Table S1 for convenience.

### Proliferation and cell cycle analysis

Cell proliferation was determined by [^3^H]-thymidine (Amersham Biosciences, NSW, Australia) incorporation as previously described [35]. The proliferation index  =  CPM of treated cells/CPM of control cells. Cell cycle analysis was performed by assessment of DNA content after staining with propidium iodide as previously described [33].

### Apoptosis and Caspase activation

Apoptosis was assessed by Annexin V/PI or 7AAD staining as previously described [35], [36]. Cells negative for annexin V and PI (or 7AAD) were considered viable. Pan-caspase activation was measured by incubation of cells with 10 µM CaspACE™ FITC-VAD-FMK (Promega) for 20 min in the dark at 37°C. Washed cells were analysis on FACSCalibur flow cytometer (Becton Dickinson) using Cell Quest software. Activation of specific caspases was assessed by flow cytometry using specific antibodies.

### Assessment of TNF-α Production

For detection of TNF-α protein, cells were treated with 2 µM monensin and 1 µg/ml Brefeldin A for 4 h at 37°C prior to fixation with Fix/Perm solution for 20 min at 4°C. Cells were washed in Perm/Wash buffer and directly labelled for TNF-α using PE conjugated anti-TNF-α antibody.

### Mitochondrial Depolarization and Cytochrome c Release

Mitochondrial depolarization was measured using 143 nM TMRM in PBS at 37°C for 20 mins, followed by labelling with annexin V Alexafluor 647 and 7AAD. A 5 min exposure to 12.5 mM CCCP (carbonyl cyanide 3-chlorophenylhydrazone) was used as a positive control. Cytochrome c release was performed using the InnoCyte Flow Cytometric Cytochrome C release Kit according to manufacturer's instructions except that goat anti-mouse Alexa Fluor 647 was used as the second layer.

### RT-PCR and qRT-PCR

Total RNA was extracted using Trizol reagent and reverse transcribed. The relative expression ratio of the target genes was compared with the housekeeping gene glyceraldehyde-3-phosphate dehydrogenase (GAPDH) using the same cDNA. Values were then normalised to the untreated sample. All samples were run in quadruplicates. The primers for SODD were: Forward: acccaagtacatatcctgtaagacc; Reverse: aggagaagcccacgcatcttc. Those for GAPDH have been previously published (Khan, et al 2008).

For the qRT-PCR cDNA was prepared as above. SODD mRNA expression levels were quantitated from standard curves generated using cDNA prepared from NALM6 cells by real-time RT-PCR using SYBR Green JumpStart Taq Ready Mix (Sigma-Aldrich, Sydney, Australia) and a Corbett 6000 thermocycler (Corbett Life Science, NSW, Australia) and specific primers: SODD forward – acatgtgctggagaaggtccagt; SODD reverse – acagagtcctggcccccagt. Values were normalised to the expression level of GAPDH using the same cDNA to generate the standard curve. The primers for GAPDH have been previously published (Bendall, et al 2004).

### Immunoprecipitation and Western blotting

Cell lysates were prepared as previously described [35]. Immunoprecipitations were performed using Protein G Dynabeads (Invitrogen) according to the manufacturer's instructions. Beads were crosslinked to antibodies to TNF-R1 (Genesearch, Arundel, QLD, Australia) or Mouse IgG2b control (DakoCytomation), using bis(sulfosuccinimidyl)suberate reagent as described by the manufacturer. Immunoprecipitated material was eluted directly into SDS-PAGE reducing buffer. Western blotting was performed as previously described [35].

### Lentiviral Knockdown of SODD

Lentiviral constructs were prepared as previously described [37] using the following shRNA sequences to SODD: SODD1 - acatatacttcatgtgtaatt or SODD2 - ccaacaatcaagatcaaagta. Lentiviral transduction was performed with Institutional Biosafety Committee approval. NALM6, LK63 or REH cells (1×10^6^/mL) were transduced in RPMI containing 10% FCS, 8 µg/ml polybrene and 40×10^6^ viral particles/ml containing shRNA for either SODD1 or SODD2 or empty vector (control) for 48 h. Viral particles were removed and the cells cultured in complete media for the indicated times. Successfully transduced cells were identified by GFP expression.

### Statistical Analysis

Comparisons between 2 groups were performed using Student's t-tests and between multiple groups using ANOVA analysis. Pairwise comparisons between groups were adjusted for multiple comparisons using Bonferroni's method. Significance was defined as having a p value of less than 0.05.

## Results

### para-NO-ASA induces apoptotic cell death in pre-B ALL cell lines

**Figure 1:**
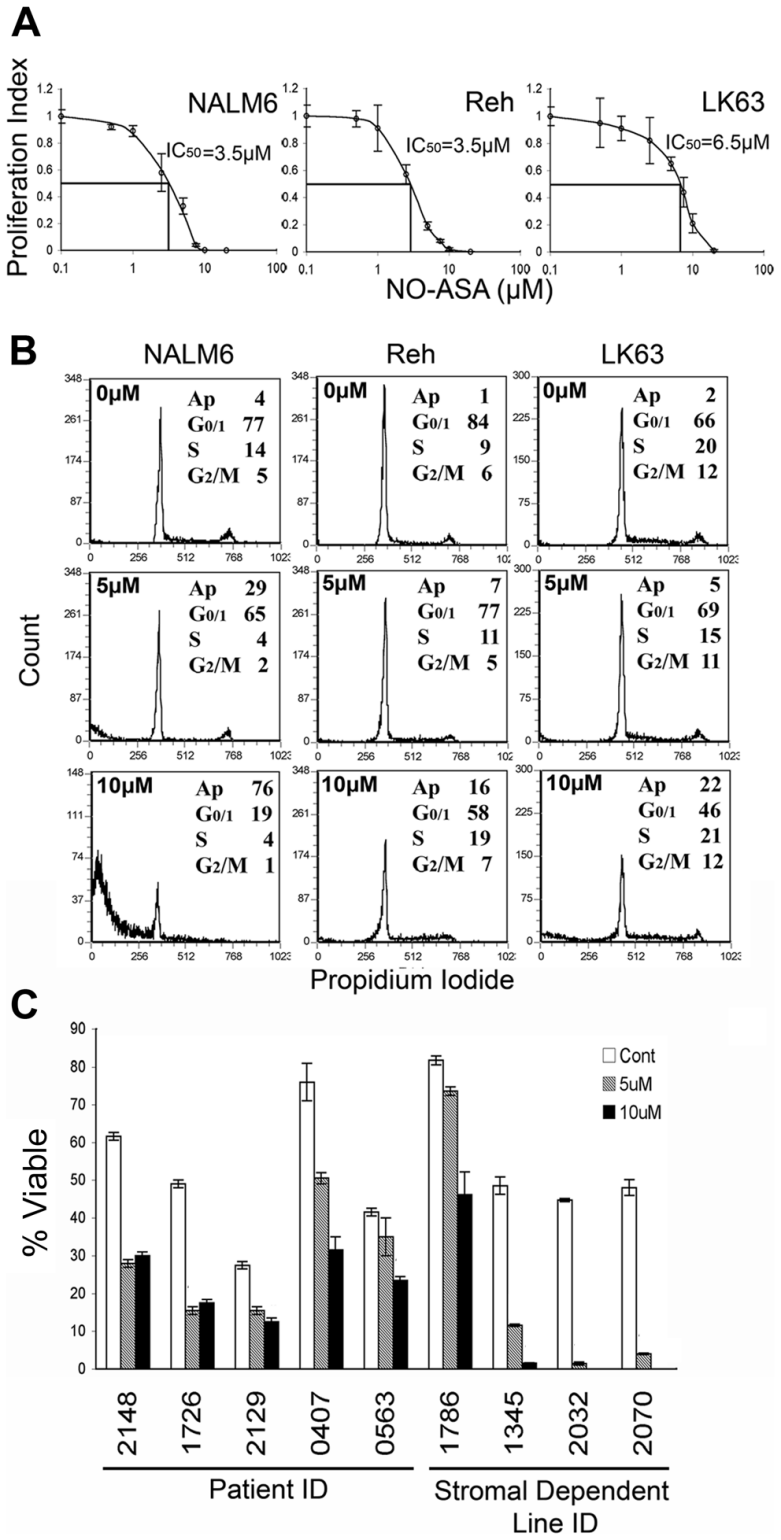
*para*-NO-ASA inhibit the proliferation and induces cell death in ALL cell lines and patient samples. (A) The proliferation of the indicated ALL cell lines as determined by ^3^H-thymidine incorporation following 24 hours incubation with *para*-NO-ASA. Vertical bars represent standard deviation of quadruplicate determinations from one of 2 independent experiments. Curves were generated using the line of best fit in Excel software. IC_50_ values are indicated. (B) Cell cycle analysis of ALL cell lines treated with indicated concentrations of *para*-NO-ASA for 24 hours. Representative plots including the mean from duplicate assessments are shown. (C) The viability of pre-B ALL patient samples or patient derived stromal dependent cell lines was assessed after 24 hours of culture in the presence of DMSO (Cont), 5 or 10 µM *para*-NO-ASA by annexin V and 7AAD staining with dual negative cells being considered viable. Vertical bars represent standard deviation of quadruplicate determinations.

**Figure 2:**
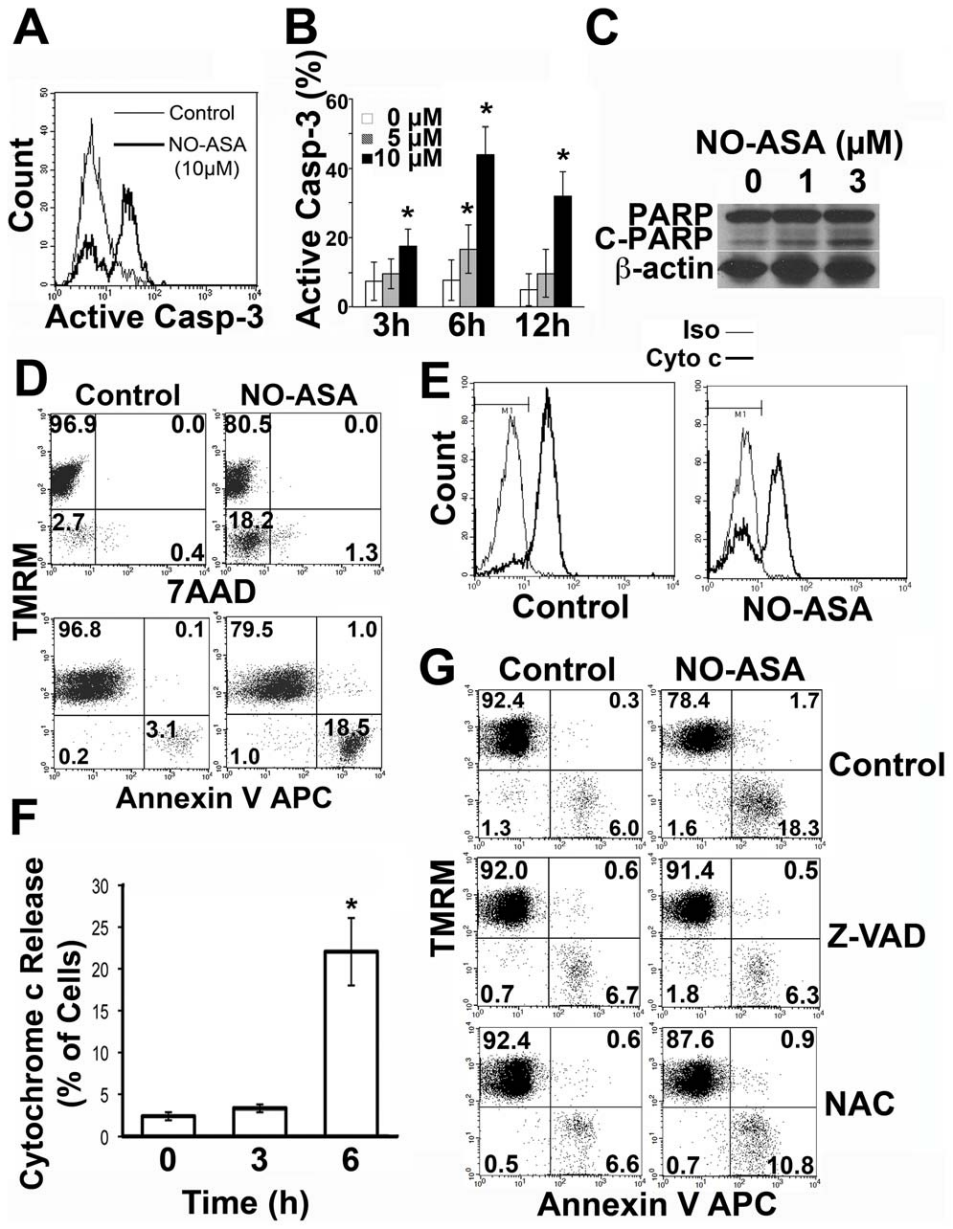
*para*-NO-ASA results in activation of executioner caspases and mitochondrial depolarization. (A) Cleavage of caspase-3 in NALM6 cells by intracellular flow cytometry following 6 hour exposure to 10 µM *para*-NO-ASA. (B) Caspase-3 activation as measured by flow cytometry, in NALM6 cells untreated or treated with 5 µM or 10 µM *para*-NO-ASA for the given time. Bars indicate the mean and s.d. of 3 independent experiments. (C) Western blot analysis of PARP activation in NALM6 cells stimulated with the indicated concentrations of *para*-NO-ASA for 12 hours. The cleaved (89 kD) and uncleaved (116 kD) forms of PARP are indicated. Data is representative of 2 independent experiments. (D) NALM6 cells were treated with 5 µM *para*-NO-ASA or vehicle for 6 hours and assessed for ΔΨ_m_ using TMRM to label cells with polarized mitochondria, and apoptosis using annexin V and 7AAD. Representative plots are shown and the mean percentage of cells from 2 experiments in each quadrant indicated. (E) Representative histograms showing cytochrome c release following exposure of NALM6 cells to 5 µM para-NO-ASA for 6 hours. The thin line represents isotype control (Iso) staining and the heavy line cytochrome c (Cyto c) staining. (F) Quantitation of cytochrome c release at the indicated time points following addition of 5 µM *para*-NO-ASA. The mean and s.d. of replicates is shown. *p = 0.02. (G) NALM6 cells were pre-treated with 100 µM Z-VAD or 2.5 mM NAC for 1 hour prior to exposure to 5 µM *para*-NO-ASA for 6 h the mean ± s.e. is shown (n≥5). *p<0.05 compared to NO-ASA alone.

We have recently shown that *para*-NO-ASA suppresses NF-κB signalling in ALL cells and that this is associated with apoptotic cell death [21]. Here we investigated the mechanism of this in more detail and extend the findings to primary patient cells. *para*-NO-ASA inhibited the proliferation of all three B cell progenitor ALL cell lines (NALM6, REH and LK63) tested in a dose dependent manner, with IC_50_ values in the low micromolar range (Fig 1A). Despite suppression of NF-κB signalling, changes in cell cycle distribution in response to *para*-NO-ASA were not consistently observed (Fig 1B and data not shown). However, a significant sub-G_0/1_ peak was detected in all three cell lines, consistent with apoptotic cell death as previously reported in ALL cell lines [21]. *para*-NO-ASA also induced cell death in 5 freshly thawed patient samples (p = 0.016 for 5 µM and 0.006 for 10 µM *para*-NO-ASA) as well as 4 patient samples that had been expanded *in vitro* by co-culture with human stromal layers (p = 0.029 for 5 µM and p = 0.0006 for 10 µM *para*-NO-ASA) (Fig 1C), demonstrating that the effect is not confined to cell lines. Closer investigation of the cell death mechanism revealed activation of the executioner caspases -3 and -7 after 12 h (Fig 2A and data not shown) with cleavage of caspase-3 in response to 5 µM and 10 µM *para*-NO-ASA in NALM6 cells (Fig 2B) showing similar kinetics to those previously reported for pan-caspase detection by Z-VAD FITC [21]. Activation of caspase-3 correlated with a dose-dependent cleavage of PARP detected by Western blotting, 12 h after drug treatment in NALM6 cells (Fig 2C). These data are consistent with our previous demonstration that *para*-NO-ASA induced cell death is caspase dependent [21].

### para-NO-ASA induces mitochondrial depolarisation (ΔΨ_m_) and mitochondrial outer membrane permeabilization (MOMP)

Loss of the inner mitochondrial transmembrane potential (ΔΨ_m_) and outer membrane permeablization (MOMP) are features of a number of cell death mechanisms including apoptotic cell death [38]. Treatment of cells with 5 µM *para*-NO-ASA resulted in a loss of ΔΨ_m_ and release of cytochrome c. Loss of ΔΨ_m_ occurred prior to the loss of plasma membrane integrity as determined by 7AAD staining (Fig 2D) and cytochrome c release (Fig 2E and F). Both occurred in close association with the induction of apoptosis as determined by annexin V staining. During cell death mediated by the intrinsic pathway, caspase activation occurs following loss of ΔΨ_m_, MOMP and cytochrome c release and formation of the apoptosome [4]. In contrast, when death is mediated via the extrinsic pathway, loss of ΔΨ_m_ and cytochrome c release is the result of caspase-8 and/or caspase-10 activation, and truncation of Bid [39]. Pan-caspase inhibition completely prevented mitochondrial depolarization implicating the extrinsic death mechanism (Fig 2G). We have previously reported that *para*-NO-ASA induced apoptosis is dependent on the generation of reactive oxygen species (ROS) [21] and here we found that inhibition of ROS by the anti-oxidant NAC also attenuated mitochondrial depolarization (Fig 2G).

### para-NO-ASA activates the initiator caspases-8, -9 and -10 but only caspase-10 activation is required for cell death

**Figure 3:**
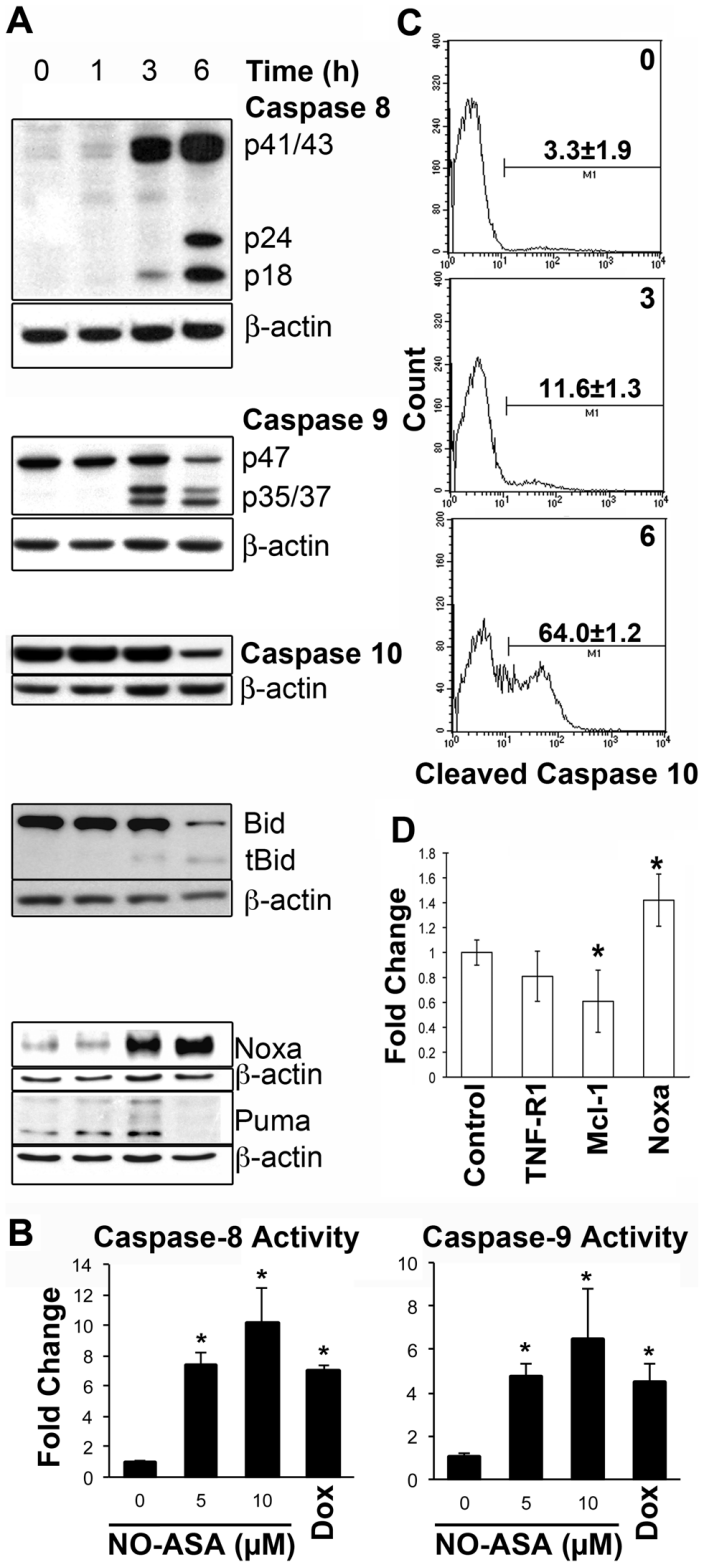
Multiple caspases are activated by *para*-NO-ASA. (A) Western blot of cell lysates from cells treated with *para*-NO-ASA for the indicated times. Western Blot of caspases-8 (cleaved forms only), -9 (full length, p47 and cleaved forms), caspase-10 (full length only), Bid (full length and cleaved forms) and full length Noxa and Puma are shown. Note that the caspase-8 antibody (#9496) only recognised the cleaved forms and not the intact protein, while the caspase-10 antibody only recognised the full-length form of the protein. (B) NALM6 cells were treated with 5 µM NO-ASA for 6 h and cell lysates analysed for caspase-8 or caspase-9 activity. The mean±SD of 3 experiments is shown. *p<0.05 compared to control. (C) Representative histograms showing *para*-NO-ASA mediated activation of caspase 10 as determined by flow cytometry. Time of exposure to *para*-NO-ASA is indicated on the histograms. The mean and s.d. from one of two independent experiments is shown. (D) Gene expression was assessed using quantitative RT-PCR in NALM6 cells treated with 10 µM *para*-NO-ASA for 12 hours. *p<0.05.

*para*-NO-ASA treatment activated the initiator caspases of the extrinsic cell death mechanism, caspase-8, and -10, as well as the initiator caspase for the intrinsic pathway, caspase-9. Cleavage of caspases-8 and -9, and loss of full-length caspase-10 following treatment with *para*-NO-ASA was demonstrated by Western blotting in NALM6 cells (Fig 3A). The activity of caspase-8 and -9 were confirmed using a colormetric activity assay (Fig 3B). The cleavage of caspase-10 could not be determined by western blotting as the antibody only recognised the full-length protein. However, activation of caspase-10 was demonstrated by flow cytometry (Fig 3C). Cleavage of all 3 caspases occurred between 1 and 3 h with no activation detectable at 1 h and some activation of all cases present at 3 h. Caspase cleavage preceded the disruption of mitochondrial function with greater than 60% of cells having activated caspase-10 by 6 h, but less than 30% of cells having disrupted mitochondria (Fig 3C&D). The percentage of cells with cleaved caspase-8 and -9 could not be determined, as Western blotting does not permit the identification of individual cells with activated caspases. Consistent with an extrinsic death mechanism Bid was processed to form tBid (Fig 3A). The presence of tBid, as well as the increase in Puma and Noxa (Fig 3A) provides a mechanism for inhibiting anti-apoptosis proteins including Bcl-2 and Bcl-X_L_ despite the lack of change in the level of these proteins (Fig S1). Furthermore, Mcl1 was down regulated in response to *para*-NO-ASA (Fig 3D). These results indicate that ultimately both intrinsic and extrinsic pathways are activated.

**Figure 4:**
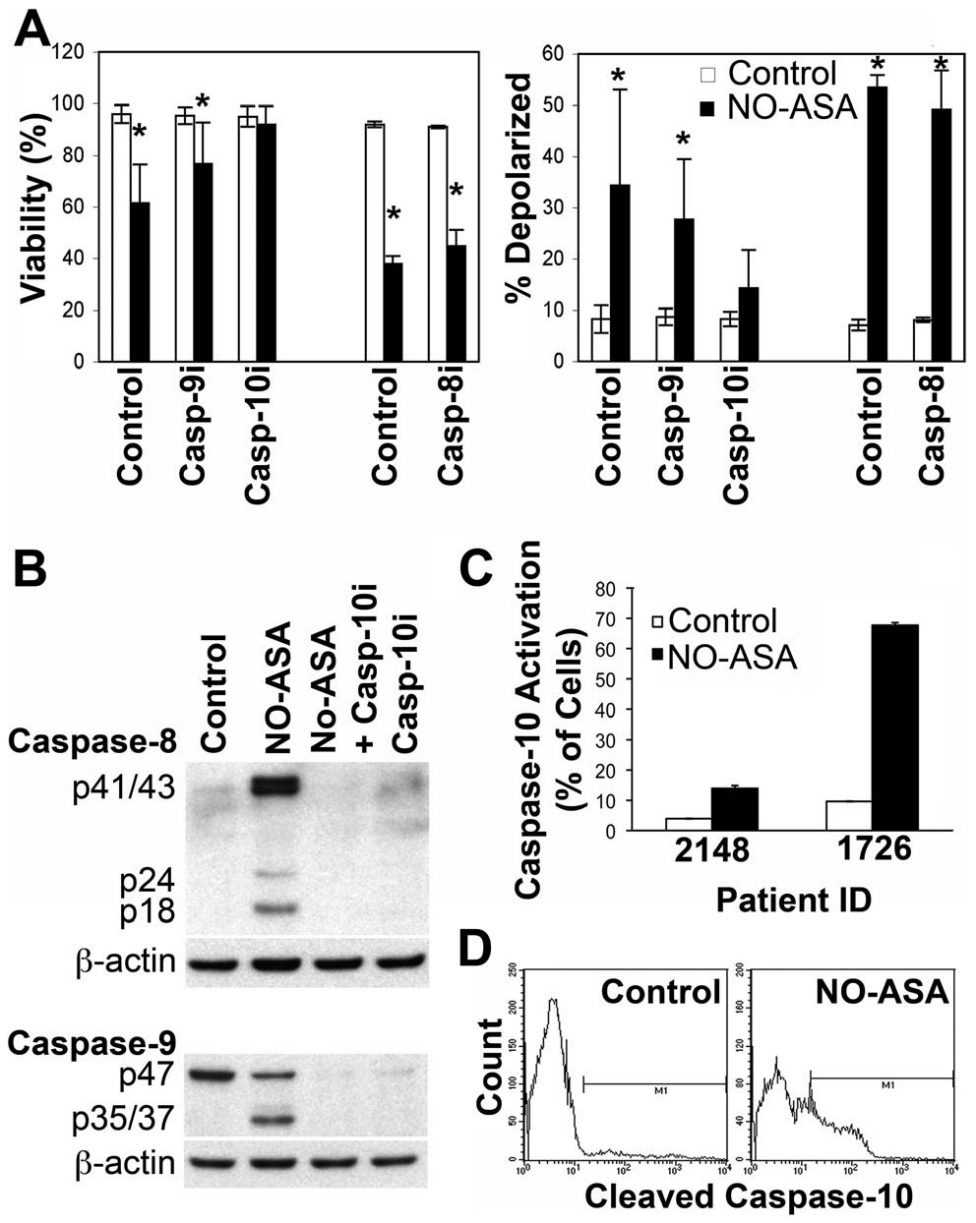
Caspase-10 activation is required for *para*-NO-ASA induced cell death. (A) The viability (top panel), as determined by 7AAD and annexin V staining, and mitochondrial depolarization (lower panel) of NALM6 cells treated with vehicle or 5 µM *para*-NO-ASA for the 30 min following pre-incubation with inhibitors of caspase-8, -9 or caspase-10. Mean and s.d. of 3 independent experiments is shown. *p<0.05. (B) Western blot showing the cleavage of caspase-8 and -9 following treatment with 10 µM *para*-NO-ASA with or without a 30 min pre-incubation with the caspase-10 inhibitor. (C) Activation of caspase-10 in patient samples exposed to 5 µM *para*-NO-ASA for 6 h as assessed by flow cytometry. The mean and s.d. of duplicate samples is shown. (D) Activation of caspase-10 in control treated NALM6 cells (left) or NALM6 cells treated with 10 µM *para*-NO-ASA (right) for 6 h. The mean and s.d. of duplicate assessments of caspase-10 activation is shown in the lower right panel.

Surprisingly inhibition of caspase-8 and/or caspase-9 failed to completely inhibit apoptosis induced by *para*-NO-ASA, although caspase-9 inhibition did have a partial but statistically insignificant effect (Fig 4A left panel and data not shown). In contrast, inhibition of caspase-10 resulted in a complete block of *para*-NO-ASA-induced apoptosis (Fig 4A). Inhibition of caspase-10 also completely prevented ΔΨ_m_ (Fig 4A right panel), suggesting that caspase-10 activation is an early and essential component of *para*-NO-ASA-mediated cell death. Indeed inhibition of caspase-10 also prevented activation of caspases-8 and -9 (Fig 4B). We have shown in Figure 1 that *para*-NO-ASA induces cell death in ALL cells from patients and as expected it also activated caspase-10 in these samples (Fig 4C).

### Caspase-10 associates with TNF-R1 following treatment with para-NO-ASA

**Figure 5:**
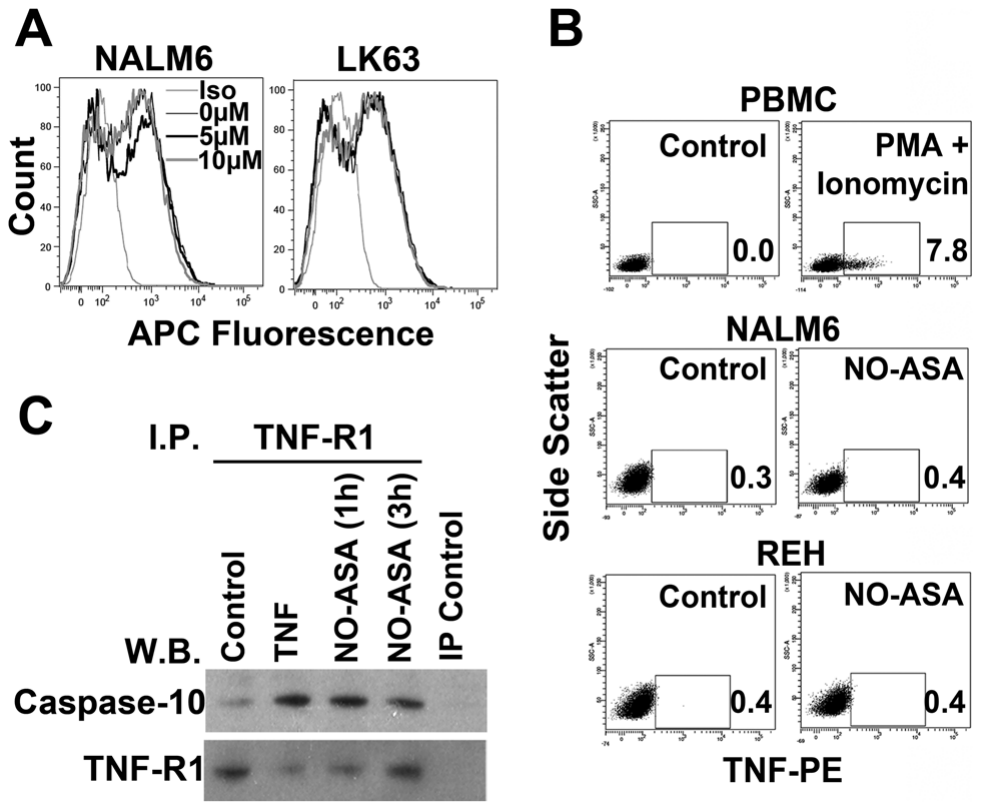
*para*-NO-ASA induces formation of the death initiating signalling complex containing caspase-10 and suppression of SODD. (A) NALM6 and LK63 cell lines were treated with 10 µM *para*-NO-ASA for 6 h and labelled for surface TNF-R1 (left panels). (B) NALM6 and REH cells were treated with *para*-NO-ASA for 6 h and assessed for TNF-α production by flow cytometry. PBMC treated with 50 ng/ml PMA and 1 µg/ml ionomycin are included as a positive control. The percentage of cells expressing TNF-α is shown on each plot. (C) NALM6 cells were treated with TNF-α (100 ng/ml), or *para*-NO-ASA (10 µM) as indicated and cell lysates prepared. TNF-R1 was immunoprecipitated and recovered complexes probed for caspase-10 or TNF-R1.

Caspase-10 forms part of the extrinsic death pathway, normally activated by factors such as TNF-α [40]. We were unable to detect any increase in TNF-R1 expression by quantitative RT-PCR (Fig 3D) or flow cytometry in response to *para*-NO-ASA (Fig 5A). Similarly there was no apparent induction of TNF-α expression in NALM6 cells following exposure to *para*-NO-ASA (Fig 5B) and there was no evidence of intracellular stores of the cytokine that could have been released following treatment. However, examination of complexes that co-precipitated with TNF-R1 demonstrated that addition of *para*-NO-ASA increases caspase-10 association with TNF-R1 (Fig 5C). We were unable to detect caspase-8 in the precipitated complexes (data not shown), however caspase-8 was also not detected in TNF-α induced complexes so it is possible this was a sensitivity issue and firm conclusions regarding the absence of caspase-8 should not be drawn from this data. Regardless, this suggests that TNF-R1 is involved in *para*-NO-ASA mediated apoptosis.

#### SODD is Over-expressed in ALL Cells and is Required for the Maintenance of Cell Viability

**Figure 6:**
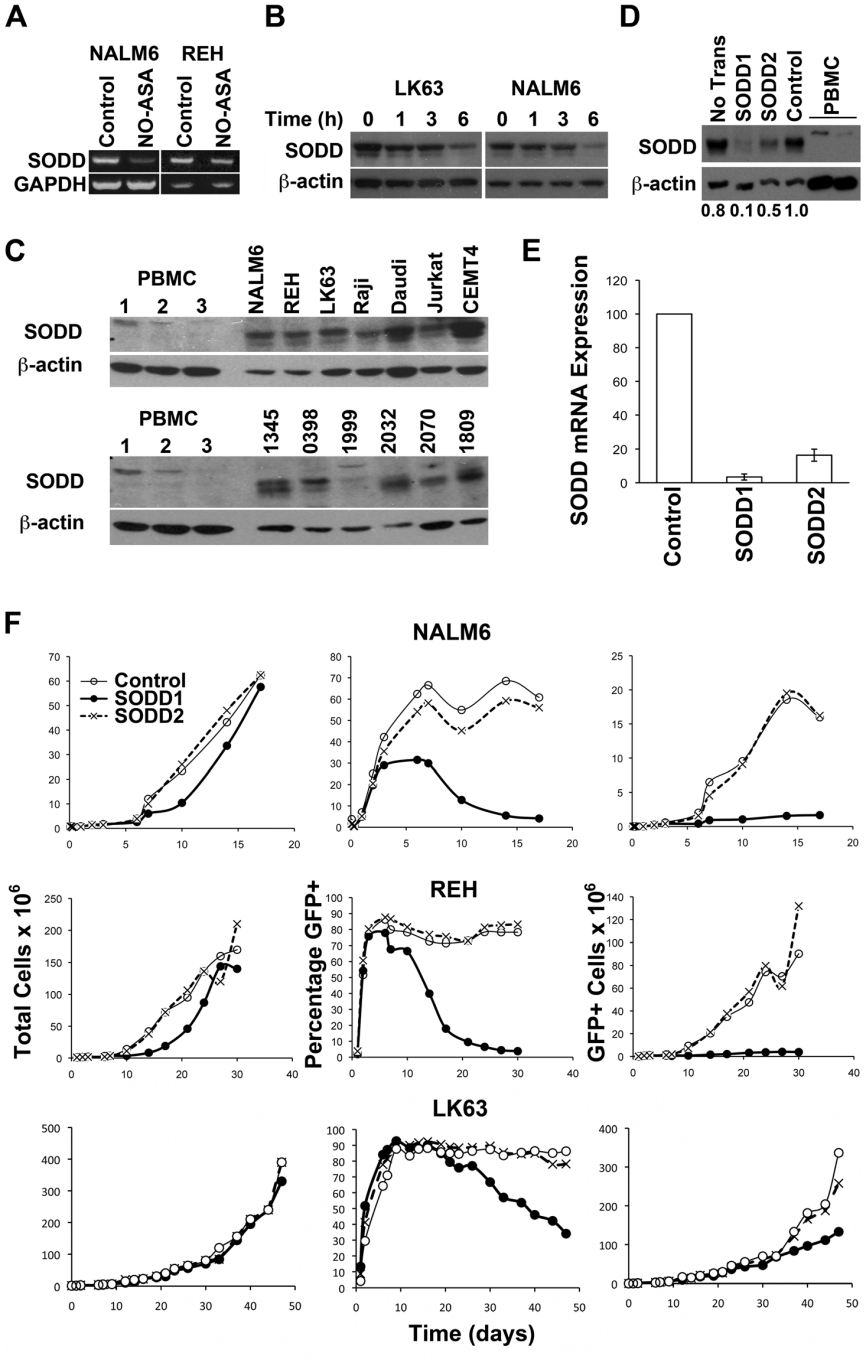
SODD is over-expressed in ALL cells and expression is required for ALL cell growth. (A) SODD expression was determined by semi-quantitative RT-PCR in NALM6 and REH cells treated with 10 µM *para*-NO-ASA for 12 h. (B) SODD expression was determined by western blotting following treatment with 10 µM *para*-NO-ASA for the indicated times. (C) Western blot analysis of SODD in normal peripheral blood mononuclear cells (PBMC), indicated cell lines (upper blots) or patient samples (lower blots). Patient samples had been expanded in NOD/SCID mice to obtain sufficient cells for Western blotting. (D) NALM6 transduced with lentiviral constructs expressing GFP alone (Control) or containing one of two shRNA specific for SODD (SODD1 and SODD2). The level of SODD protein (D) was determined in LK63 cells after 44 days in culture while mRNA levels were assessed in NALM6 cells on day 6 of culture by qRT-PCR and was normalised to the levels of GAPDH (E). The mean and s.e.m. of three independent experiments is shown. (F) The total cell number (left panels), and the percentage (centre panels) and total number of GFP+ transduced cells (right panels) was monitored over time. The mean and s.d. of duplicate cultures is shown.

SODD is thought to prevent spontaneous, and suppress TNF-α-induced, trimerisation of TNF-R1 and signalling [6], [7], and is also reportedly over-expressed in ALL cells [41], a finding which we have confirmed and extended here (Fig 6C). Interestingly the smaller isoform was predominant in leukemic cells, while the larger isoform was more abundant in normal peripheral blood mononuclear cells (PBMCs), although both forms were present in normal PBMC on longer exposure. *para*-NO-ASA resulted in down regulation of SODD mRNA and protein levels (Fig 6A&B). Consistent with SODD preventing activation of TNF-R1, ALL cells were found to be refractory to cell death induced by exogenous TNF-α (Fig S2). In contrast, a small but significant proliferative response to TNF-α was detected, probably via activation of TNF-R2 (Fig S2). Lentiviral based constructs to knockdown of SODD were prepared, and although both significantly reduced SODD expression, the first construct, SODD1, was superior, producing a log greater reduction in SODD mRNA expression in NALM6 cells on day 6 of the culture than SODD2 (Fig 6E): similar results were obtained for REH cells. In LK63 cells sufficient transduced cells were obtained to examine the knockdown of the SODD protein. Here the SODD1 construct was also superior to SODD2 (Fig 6D), although neither reduced SODD to the level seen in normal peripheral blood cells (Fig 6D). Culture of NALM6, REH and LK63 cells following infection with the control and SODD2 lentiviral constructs resulted in the expected cell expansion, but those transduced with SODD1 showed significant growth retardation (Fig 6F, left panels). The transduction efficiency in cells transduced with control and SODD2 constructs was high and sustained over time, while that in cells transduced with the SODD1 construct was significantly lower, resulting in a major reduction in the number of GFP expressing cells in these cultures (Fig 6F, centre and left panels). In LK63 cells, where the culture time was further extended, the cells expressing the SODD2 construct also started to decline in the culture, albeit at a slower rate. This suggests that a threshold level of SODD is required for the survival of ALL cells and by inference that *para*-NO-ASA induced cell death is at least partially the result of the reduction of SODD expression.

## Discussion

We report here that *para*-NO-ASA results in apoptotic cell death mediated through activation of caspase-10. The biochemical process appears to relate closely to death receptor mediated apoptosis, with activation of caspase-10 being the initiating event followed by cleavage of Bid, and loss of ΔΨ_m_ and MOMP. Ultimately the intrinsic death mechanism is triggered with activation of caspase-9, and the executioner caspases-3 and -7. Cleavage of caspase-10 was subsequent to its association with TNF-R1. This demonstrates that *para*-NO-ASA is mediating cell death via activation of the extrinsic cell death mechanism.

Caspase-8 plays the predominant role in most scenarios involving extrinsic cell death, including those triggered by TNF-α. In contrast, *para*-NO-ASA resulted in caspase-10 dependent death in ALL cells with no measurable role for caspase-8. Caspase-10 is highly expressed in lymphoid cells and is frequently down regulated in lymphoid cell lines [40], and other cancers [42], [43]. Mutations in caspase-10 are associated with autoimmune lymphoproliferative syndrome type II, suggesting that caspase-10 is important for the regulation of normal lymphoid cells [15]. Although mutations in caspase-10 have been demonstrated in Non-Hodgkin's lymphoma and are thought to contribute to disease pathogenesis [16], such mutations have not been detected in B lineage ALL [17], and are not associated with breast or ovarian cancer risk [44]. It is therefore likely that the majority of ALL samples will have functional caspase-10. Cell death that is exclusively or predominantly caspase-10 dependent has only rarely been reported, even within lymphoid populations. In most of these settings, caspase-10 activation occurred downstream of the mitochondria and independently of death receptors. Examples include cell death induced by Taxol in the human lymphoblastic leukemia cell line, CCRF-HSB-2 [20], arsenic trioxide in acute promyelocytic leukemia cells [19], etoposide in U937 cells [45] and a number of other cell lines including Jurkat T cells [46], as well as in a colon cancer model [43]. In our study there was clear association between caspase-10 and TNF-R1, which preceded caspase activation, suggesting that TNF-R1 plays an active role in the process. However our inability to perform gene knockdown studies for caspase-10 and potential off target effects of the caspase-10 inhibitor used leaves room for the role of other caspases in *para*-NO-ASA induced cell death.

How the extrinsic death mechanism is triggered by *para*-NO-ASA is not entirely clear. B-lineage ALL cells only rarely express TNF-α [10], [47], and we were unable to demonstrate any background, or *para*-NO-ASA-induced TNF-α production by NALM6 cells. This suggests that activation of TNF-R1 may be occurring in a ligand independent manner. Death receptors including TNF-R1 are known to self-aggregate, particularly when over-expressed and SODD is believed to play a role in keeping this in check [6]. Ligand-independent association has been previously reported in lung and breast carcinoma and although associated with DNA damage, DNA damage alone was not sufficient to activate TNF-R1 [48]. However the possibility remains that TNF-α, below the limit of detection, may be present and activates TNF-R1. We were unable to detect evidence of *para*-NO-ASA induced DNA damage using γ-H2AX staining prior to apoptosis driven DNA fragmentation (data not shown). This suggests that the DNA damage response is not involved. More recently ligand-independent activation of TNF-R1 has been associated with oxidative conditions and modification of cysteine residues within the extracellular cysteine rich domains of TNF-R1 in the model cell systems of renal and cervical cancer, most likely in the pre-ligand assembly domain (PLAD) [49]. This may be important in *para*-NO-ASA-induced cell death, as we have previously shown that reactive oxygen species are produced and are required for cell death in this setting [21].

Another possibility is that suppression of SODD by *para*-NO-ASA permits constitutive activation through TNF-R1 self-association. SODD is an anti-apoptotic protein that associates with the death domain of TNF-R1, Fas and death receptor-3 (DR3), and thereby negatively regulates cell death signalling [6], [7]. The importance of SODD in maintaining TNF-R1 in an inactive conformation under physiological conditions has not been fully resolved, with one study finding no phenotype in SODD deficient mice, while another reported enhanced cytokine production [50], [51]. Here we observed loss of SODD concomitantly with caspase-10 association with TNF-R1, which is consistent with SODD playing an active role in keeping TNF-R1 signalling in check. SODD is also reportedly over expressed in ALL [41], a finding that we confirmed in this study.

**Figure 7:**
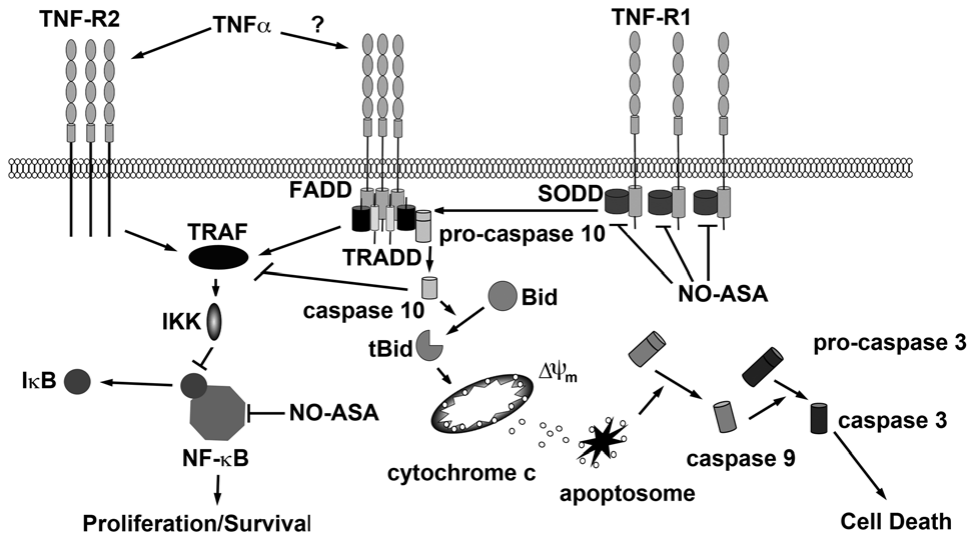
Schematic diagram illustrating the proposed mechanism of action of *para*-NO-ASA. *para*-NO-ASA down regulates SODD allowing self-aggregation, or enhancing TNF-α-induced, activation of signalling through TNF-R1. TNF-R1 signalling triggers the extrinsic apoptosis cascade including cleavage of pro-caspase-10 and Bid, mitochondrial depolarization, outer membrane permeabilization and release of cytochrome c. This is followed by cleavage of pro-caspase-9 and the executioner caspases-3 and -7. While signalling through TNF-R1 can activate NF-κB this may be suppressed by caspase-mediated cleavage of TRAF. In contrast, TNF-R2 signal induces cell survival and proliferation pathways, predominantly through NF-κB but also MAPK signalling. In ALL cells where SODD is over expressed, exogenous TNF-α is likely to predominantly signal through TNF-R2 resulting in increased survival and proliferation. Activating TNF-R1 by down regulation of SODD provides a mechanism for inducing cell death without increasing proliferation and survival signals. FADD, Fas-associated death domain; IKK, inhibitor of κB kinase; I-κB, inhibitor of κB; TRADD, TNF-R1-associated death domain; TRAF, TNF receptor-associated factor.

The death inducing ligands, TNF-α, Fas and TRAIL have not demonstrated the hoped for potency in killing malignant cells and methods to enhance the efficacy of these agents have been explored with varying degrees of success [52], [53]. The role of TNF-α in ALL cell biology is unclear with the initial report showing suppression of ALL cell proliferation [47], while subsequent reports suggest that TNF-α enhances ALL cell growth [54]. The co-expression of TNF-R2, which lacks death domains in its cytoplasmic tail and hence only activates pro-survival NF-κB signalling [55], would also be expected to promote proliferation and survival in ALL cells. The pro-survival signals activated by NF-κB are known to limit the cytotoxic effects of TNF-α and other death ligands [56]. Over expression of SODD would be expected to further enhance ALL cell survival by preventing signalling through TNF-R1 and other death receptors such as Fas and DR3, but not TNF-R2 [6], [41]. It is tempting to speculate that the potency of *para*-NO-ASA results from activation of signalling through TNF-R1, due to suppression of SODD, in the absence of any increase in NF-κB signalling through TNF-R2 (Fig 7). Furthermore, we have previously shown that *para*-NO-ASA suppresses NF-κB signalling [21]. Therefore, unlike TNF-α, *para*-NO-ASA activates death receptor signalling by inhibition of SODD while simultaneously suppressing pro-survival NF-κB signalling. We were surprised to discover that suppression of SODD expression alone was sufficient to significantly impair the growth of ALL cell lines. This finding is reminiscent of cells displaying oncogene addiction, which are killed following oncogene withdrawal, while normal cells are relatively unaffected. This suggests that targeting SODD may be a new therapeutic strategy for the treatment of ALL and other malignancies where SODD is overexpressed.

## Supporting Information

Figure S1 Flow cytometric analysis of Bcl-X_L_ and Bcl-2.
(DOCX)

Figure S2 Expression of TNF-R2 and effect of TNF-α on ALL cell lines.
(DOCX)

Table S1 Patient Information.
(DOCX)
