# Supplementary material for: Silencer of Death Domains Controls Cell Death through Tumour Necrosis Factor-Receptor 1 and Caspase-10 in Acute Lymphoblastic Leukemia
Source: PLoS One. 2014 Jul 25;9(7):e103383. doi: 10.1371/journal.pone.0103383 (PMC4111576; doi:10.1371/journal.pone.0103383)
Supplement: Figure S1 — Flow cytometric analysis of Bcl-XL and Bcl-2. (DOCX) [file pone.0103383.s001.docx]

**
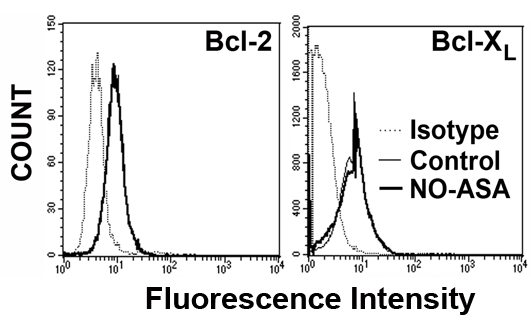
**

**Figure S1**

Flow cytometric analysis of NALM6 cells treated with vehicle or 5 µM *para*-NO-ASA for 6 h. Overlay histograms include isotype control stained cells (Isotype) and specific antibody stained cells treated with *para*-NO-ASA (NO-ASA) or vehicle (Control) as indicated.
