# Supplementary material for: Silencer of Death Domains Controls Cell Death through Tumour Necrosis Factor-Receptor 1 and Caspase-10 in Acute Lymphoblastic Leukemia
Source: PLoS One. 2014 Jul 25;9(7):e103383. doi: 10.1371/journal.pone.0103383 (PMC4111576; doi:10.1371/journal.pone.0103383)
Supplement: Figure S2 — Expression of TNF-R2 and effect of TNF-α on ALL cell lines. (DOCX) [file pone.0103383.s002.docx]

**
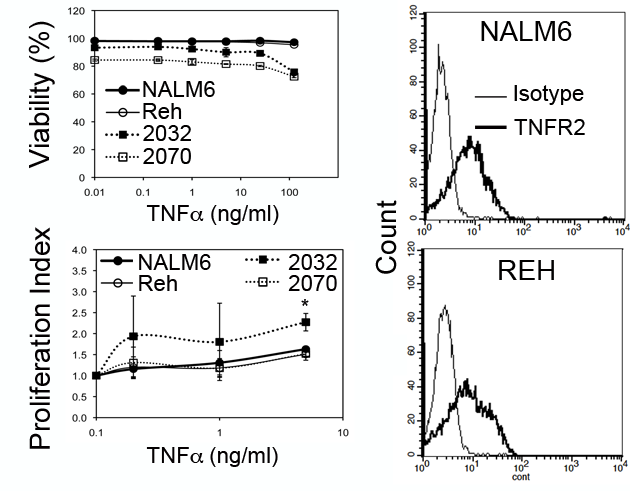
**

**Figure S2**

The indicated cell lines were treated with specified concentrations of TNF-α for 72 h and analysed for survival using annexin V/PI staining by flow cytometry (upper left panel) or proliferation using [^3^H]-thymidine incorporation (lower left panel). The expression of TNF-R2 was assessed by flow cytometry (right panels).
