# Supplementary material for: Silencer of Death Domains Controls Cell Death through Tumour Necrosis Factor-Receptor 1 and Caspase-10 in Acute Lymphoblastic Leukemia
Source: PLoS One. 2014 Jul 25;9(7):e103383. doi: 10.1371/journal.pone.0103383 (PMC4111576; doi:10.1371/journal.pone.0103383)
Supplement: Table S1 — Patient Information. (DOCX) [file pone.0103383.s003.docx]

**Table S1. Patient Information**

| Patient ID | Age/Sex | Immuno-phenotype | Cytogenetics |
| --- | --- | --- | --- |
| Uncultured Samples | | | |
| 2148 | 6/F | CD34^+^CD10^+^ | N/A |
| 1726 | 57/M | CD34^+^CD10^+^ | N/A |
| 2129 | 6/M | CD34^+^CD10^+^ | N/A |
| 0407 | 45/M | CD34^-^CD10^+^ | t(1;19)^1^ |
| 0563 | 2/M | CD34^-^CD10^+^ | N/A |
| Samples Expanded on Stroma | | | |
| 1786 | 12/F | CD34^-^CD10^+^ | No metaphases |
| 1345 | 5/F | CD34^-^CD10^+^ | 45XX dup(1)(q42q25),del(3)(q21),-9,del(9)(p22) t(18;20)(q21;13.1) |
| 2032 | 12/M | CD34^+^CD10^+^ | 46 XY add (9)(p24), del(9)(p21), del(13)(q11q21), der(19) t(1;19)(q23;p13) |
| 2070 | 65/M | CD34^+^CD10^+^ | 45 XY t(9;22) (q34;q11.2) del(9) (p21) |

^1^ Determined by PCR.
